# Editorial: Sarcoidosis diagnosis and treatment based on etiology

**DOI:** 10.3389/fmed.2026.1802542

**Published:** 2026-03-03

**Authors:** Michiru Sawahata, Yoshinobu Eishi

**Affiliations:** 1 Department of Respiratory Medicine, Jichi Medical University, Shimotsuke, Tochigi, Japan; 2 Department of Human Pathology, Graduate School of Medical and Dental Sciences, Institute of Science Tokyo, Tokyo, Japan

**Keywords:** biomarker, diagnosis, etiology, sarcoidosis, treatment

Sarcoidosis is a heterogeneous granulomatous disease with variable organ involvement, clinical course, and treatment response. Because the etiology is often uncertain at the point of care, clinicians must work with probabilistic, testable “working” etiological hypotheses—explicitly ranking plausible triggers/exposures and updating them as new data become available—to refine diagnosis, exclude mimics, and choose therapy. This Research Topic, “*Sarcoidosis diagnosis and treatment based on etiology*,” brings together 10 contributions that collectively support an etiology-informed, precision-management pipeline, summarized in Figure 1.

**Figure 1:**
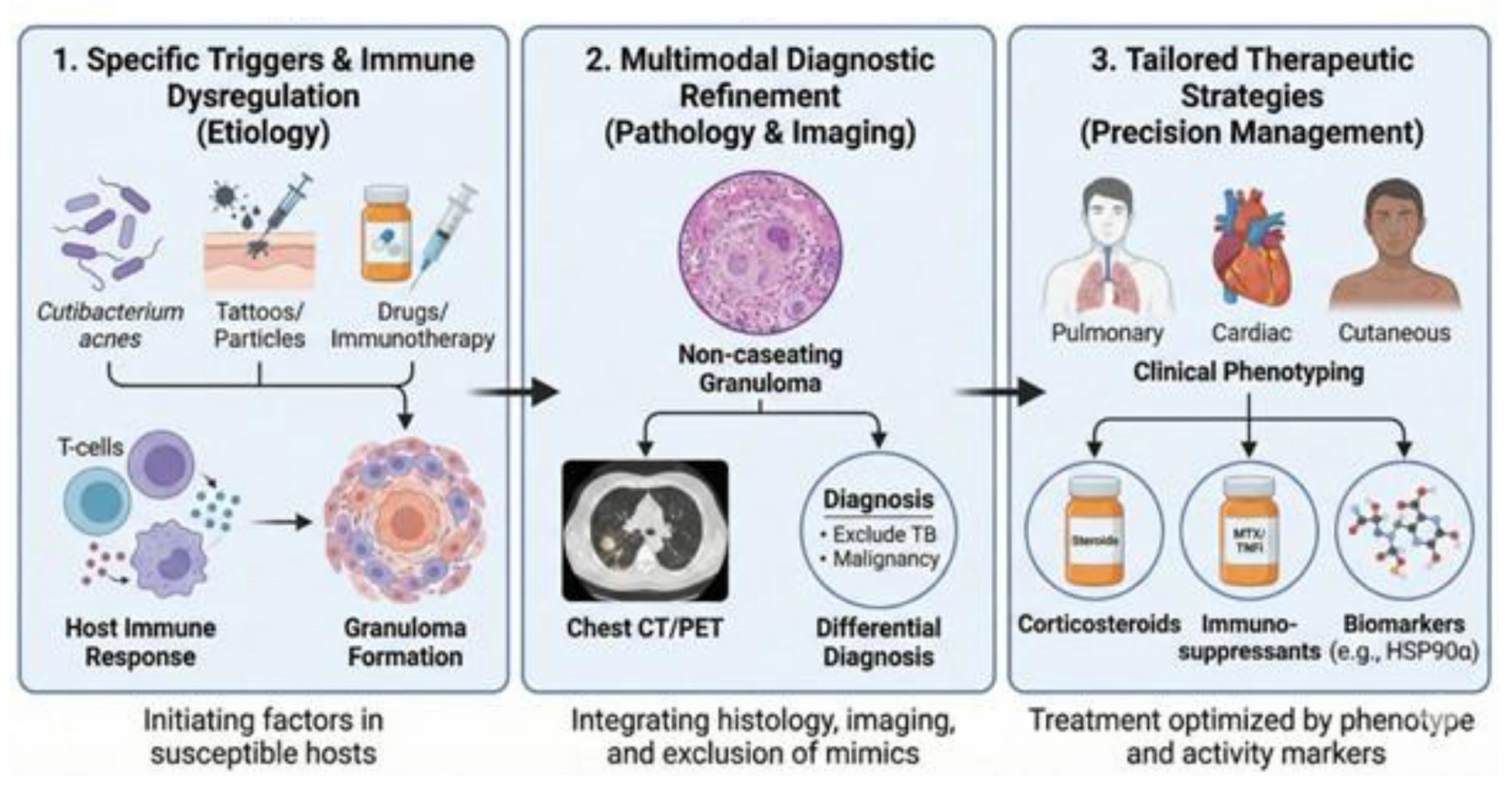
Etiology-based precision management pipeline for sarcoidosis. The flowchart outlines a three-step framework: (1) specific (candidate) triggers and host immune dysregulation (including Cutibacterium/Propionibacterium acnes, tattoos/particles, and drugs/immunotherapy) leading to granuloma formation; (2) multimodal diagnostic refinement integrating pathology and imaging (e.g., non-caseating granuloma with chest CT and PET where appropriate) and a structured differential diagnosis to exclude key mimics (e.g., tuberculosis and malignancy); in practice, other granulomatous mimics such as chronic beryllium disease and CVID-associated granulomatous disease should also be considered when clinically indicated; and (3) tailored therapeutic strategies guided by clinical phenotyping (e.g., pulmonary, cardiac, and cutaneous disease), including corticosteroids, immunosuppressants (e.g., methotrexate and TNF inhibitors), and activity/biomarker assessment (e.g., HSP90α). Routine monitoring typically integrates symptoms, pulmonary function, imaging, and conventional serological indicators such as ACE and soluble IL-2 receptor.

Operationalizing a “working” etiological hypothesis means translating mechanistic uncertainty into a structured bedside workflow. When direct evidence of a trigger (e.g., Cutibacterium acnes or tattoo particles) is absent, clinicians can: (i) systematically document exposure and treatment history (including tattoos/implants, occupational or environmental metal exposure such as beryllium exposure, drugs and immune checkpoint inhibitors, and travel/zoonotic risks); (ii) seek supportive—but imperfect—signals from phenotype and tests (e.g., organ distribution, radiologic patterns, bronchoalveolar lavage profiles, histologic context, targeted microbiology, and, where available, immunohistochemistry/PCR for candidate antigens); and (iii) iteratively revise the hypothesis after high-consequence mimics are excluded and longitudinal response is observed. In this sense, “etiology” functions as a Bayesian prior that is refined by pathology, imaging, and activity markers rather than as a binary label.

Beyond its clinical heterogeneity, sarcoidosis can be conceptually understood as an antigen-driven, immune-mediated syndrome that occupies a distinct position between classical infection and autoimmunity, arising from a failure of antigen-specific immune tolerance to persistent antigens rather than from uncontrolled pathogen proliferation or primary autoimmunity.

## Aims of the Research Topic

The Research Topic was designed to (i) highlight putative triggers and host immune dysregulation, (ii) strengthen multimodal diagnostic refinement through pathology and imaging while rigorously excluding major mimics (notably tuberculosis and malignancy), and (iii) connect clinical phenotyping to tailored therapeutic strategies and activity markers.

## From triggers to granuloma formation (step 1)

Two reviews set the conceptual foundation: “*A review of sarcoidosis etiology, diagnosis and treatment*” (Waly et al.) synthesizes current knowledge across etiological hypotheses, diagnostics, and management, while “*Diagnosis and treatment of sarcoidosis based on immunological etiology and mechanisms*” (Zhao et al.) focuses on immunological endotypes that may underlie clinical heterogeneity. Mechanism-to-therapy translation is further developed in “*Latent microbial reactivation and immune dysregulation in sarcoidosis: bridging pathogenesis and precision therapeutics*” (Sawahata, Uchida et al.), which frames microbial/antigen hypotheses and immune dysregulation as an actionable clinical context for treatment decisions. Etiology-oriented case evidence is provided in the “*Case Report: Tattoo sarcoidosis with epithelioid cell granuloma positive for Propionibacterium acnes*” (Masuda et al.), which supports the relevance of Cutibacterium (formerly Propionibacterium) as a candidate antigen in susceptible hosts, and in the “*Case Report: The immune architecture of immunotherapy-induced cutaneous sarcoidosis resembles peritumoral inflammation*” (Wang et al.), illustrating treatment-associated triggers and immune perturbation.

## Multimodal diagnostic refinement (step 2)

The pipeline's diagnostic core integrates histology (non-caseating granuloma), imaging (including CT and FDG-PET where appropriate), and systematic exclusion of sarcoidosis mimics. The article “*Pathological diversity of pulmonary sarcoidosis*” (Takemura) details the spectrum of histopathological patterns that can inform clinically meaningful phenotyping, while the “*Case Report: Sarcoidosis or tuberculosis? A continuous challenge*” (Strambu and Beer) emphasizes the enduring necessity of differential diagnosis—particularly TB exclusion—before immunosuppression. Beyond the mimics discussed in this Research Topic (e.g., tuberculosis and malignancy), clinicians should also remain vigilant for other clinically important granulomatous mimics, such as chronic beryllium disease (CBD) and common variable immunodeficiency (CVID)-associated granulomatous disease (e.g., granulomatous-lymphocytic interstitial lung disease), both of which can present with non-caseating granulomas and multisystem involvement. These entities are best addressed through targeted exposure and infection histories, and focused testing when appropriate (e.g., beryllium sensitization testing, such as the BeLPT, and serum immunoglobulin evaluation). This approach helps to avoid misclassification and to align treatment with the underlying pathobiology.

## Tailored therapeutic strategies and monitoring (step 3)

Precision management depends on linking phenotype (e.g., pulmonary, cardiac, or cutaneous) with treatment intensity and monitoring. “*Clinical characteristics of patients with pulmonary sarcoidosis treated with systemic steroids in Japan*” (Sawahata, Kimura et al.) provides practice-oriented data on steroid-treated cohorts, and “*Phenotyping sarcoidosis: a single institution retrospective analysis*” (Bertuccio et al.) illustrates how structured phenotyping can stratify patients with differing trajectories. “*Upregulation of HSP90*α *in the lungs and circulation in sarcoidosis*” (Isshiki et al.) adds biomarker-oriented insights that may complement clinical activity assessment and guide follow-up. In routine monitoring, serum ACE levels and soluble IL-2 receptors (sIL-2R) remain widely used indicators of granulomatous activity, but both have recognized limitations in specificity and can be influenced by host factors and comorbidities. Although not the primary focus of the included HSP90α study, HSP90α may represent a complementary axis of immune/stress activation, and a pragmatic near-term approach is to integrate it (where available) as an add-on marker alongside symptoms, pulmonary function tests, imaging, and established serological tests—pending further validation and assay standardization.

## Future directions

Across the Research Topic, a consistent message emerges: “etiology” should be treated as a testable clinical hypothesis that is iteratively refined by pathology, imaging, and exposure/treatment history, then operationalized through phenotype-guided therapy and biomarkers. Prospective studies that couple standardized phenotyping with etiology-informed treatment strategies and that validate activity markers will be essential to advancing individualized care. In summary, this Research Topic provides a coherent precision-management pipeline—from putative triggers to diagnosis, phenotyping, therapy, and monitoring—to support better decision-making for patients with sarcoidosis.

From a conceptual standpoint, future progress will depend on clarifying how persistent antigens, host immune tolerance failure, and tissue-specific immune architecture interact to generate distinct sarcoidosis phenotypes.

